# Role of Hyperintense Acute Reperfusion Marker for Classifying the Stroke Etiology

**DOI:** 10.3389/fneur.2017.00630

**Published:** 2017-11-29

**Authors:** Hee Young Choi, Kyung Mi Lee, Hyug-Gi Kim, Eui Jong Kim, Woo Suk Choi, Bum Joon Kim, Sung Hyuk Heo, Dae-Il Chang

**Affiliations:** ^1^Department of Radiology, Kyung Hee University College of Medicine, Kyung Hee University Hospital, Seoul, South Korea; ^2^Department of Neurology, Kyung Hee University College of Medicine, Kyung Hee University Hospital, Seoul, South Korea

**Keywords:** hyperintense acute reperfusion marker, post-contrast fluid-attenuated inversion recovery, acute stroke, Trial of ORG 10172 in Acute Stroke Treatment classification, magnetic resonance imaging

## Abstract

**Purpose:**

The hyperintense acute reperfusion marker (HARM) is a delayed enhancement of the subarachnoid or subpial space observed on post-contrast fluid-attenuated inversion recovery (FLAIR) images and is associated with permeability changes to the blood–brain barrier in acute stroke. We investigated the relationship between HARM and stroke etiology based on the Trial of ORG 10172 in Acute Stroke Treatment (TOAST) classification. In addition, we evaluated the relationship between HARM and stroke locations with respect to vascular territories and anatomic compartments.

**Materials and methods:**

We recruited 264 consecutive patients (109 women; mean age 68.63 years) who were diagnosed with acute ischemic stroke and underwent brain magnetic resonance imaging (MRI) including post-contrast FLAIR and DWI within 7 days of symptom onset from May 2015 to March 2016 for this retrospective study. Post-contrast FLAIR images were obtained 5 min after gadolinium administration. The mean time interval between the onset of stroke symptoms and MRI acquisition in total included patients was 18 h and 7 min (median 12 h and 57 min, range 2–127 h). We analyzed the overall incidence and distribution patterns of HARM in acute ischemic stroke cases and compared the relative incidence and distribution patterns of HARM between the subgroups of stroke etiology based on conventional TOAST classification. We obtained odds ratio (OR) of HARM in different stroke locations based on vascular territories and anatomical compartments. This study was approved by our institutional review board.

**Results:**

Among the 264 patients, 67 (25.38%) patients were HARM positive and 197 (74.62%) patients were HARM negative. There was significant difference in HARM incidence among the stroke subgroups (*p* < 0.001). Small vessel occlusion (SVO) was associated with the HARM-negative group (*p* < 0.001), while large artery atherosclerosis (LAA) and cardioembolism (CE) were associated with the HARM-positive group (*p* = 0.001). Also, regional pattern of HARM on the same vascular territory as the acute infarction was dominantly demonstrated regardless of stroke etiology. The OR for HARM from middle cerebral artery (MCA) infarction was 1.868 [95% confidence interval (CI): 1.025–3.401]. The OR for HARM from cortical infarction was 9.475 (95% CI: 4.754–18.883) compared to other anatomic compartments.

**Conclusion:**

The presence of the HARM was significantly associated with embolic infarctions including LAA and CE. Conversely, SVO was exclusively associated with the absence of the HARM. Second, MCA and cortical infarction showed a more pronounced HARM compared to infarctions at other vascular territories and anatomic compartments. According to the results in the current study, we speculate that the presence of HARM on post-contrast FLAIR images was associated with specific stroke causes especially in embolic causes.

## Introduction

Stroke is one of the main causes of death and disability in the world. In the USA, stroke has been one of the leading causes of death since 1924 (1). The global population comprised a significant number of elderly individuals, causing an increased incidence of age-related diseases such as stroke. Therefore, an accurate definition of the mechanism of stroke is crucial, as this will guide the selection of the most effective care and therapy (2, 3). There are many factors contributing to the overall outcome and prognosis in stroke patients. In addition to other predictors of stroke prognosis, such as neurological severity, age, other comorbidities, and epidemiological factors, the etiological subtype in ischemic stroke is an important factor for predicting the prognosis. For example, patients with lacunar infarcts have a better prognosis up to 1 year after onset than other stroke subtypes due to a different pathophysiological mechanism, although the long-term prognosis after lacunar stroke may not greatly differ from that after non-lacunar stroke. In contrast, patients with stroke of cardioembolic (CE) or large artery atherosclerosis (LAA) etiology tend to have worse prognoses for recovery than those with other ischemic stroke subtypes (4, 5). In this context, it is important to appropriately diagnose the patient’s etiological subtype of ischemic stroke not only for assisting treatment decisions by physicians but also for designing strategies for secondary stroke prevention (6).

There are several etiologic classification systems for ischemic stroke, such as the Harvard Stroke Registry (7), Stroke Data Bank (8), Trial of ORG 10172 in Acute Stroke Treatment (TOAST) system (9), Baltimore-Washington system (10), Causative Classification of Stroke System (CCS) (11), and atherothrombosis, small vessel disease, cardiac causes, and other uncommon causes (12). These are further subcategorized into the following two types according to the major approaches for the etiologic classification of stroke: phenotype and causative types. The phenotypic subcategory is determined based on an organization of abnormal test findings that may fit into major etiologic groups. Using this classification, patients could be categorized into more than one etiologic subtype. In contrast, the causative subcategory is assigned according to the most suspicious presumed mechanism of stroke. TOAST and CCS are the most commonly available examples of causative systems. However, with recent advances in diagnostic technology, multiple coexisting potential causes of ischemic stroke are frequently identified, which makes the selection of a causative subgroup a subjective process in clinical practice.

The TOAST classification system is primarily based on clinical features and also incorporates diagnostic information from various modalities including computed tomography, magnetic resonance imaging (MRI), transthoracic echocardiography, extracranial carotid ultrasonography, and cerebral angiography. This classification includes five subtypes of cerebral infarction: (1) LAA, (2) small vessel occlusion (SVO), (3) cardioembolism (CE), (4) strokes of other determined etiology (SOD), and (5) strokes of undetermined etiology (SUD) (Table 1). The SUDs are further sub-classified into “two or more causes,” “negative evaluation,” and “incomplete evaluation” (9). Although the TOAST system has been widely used in stroke-related research and in therapeutic studies, it has a few weaknesses. First, the reliability of TOAST classification is only fair to moderate with kappa values ranging from 0.42 to 0.54 (9, 13–16). Second, a result of “undetermined etiology” is often reached, partially because patients with more than two causes of stroke are classified as “undetermined etiology,” even though the individual potential causes of stroke are identifiable (6, 11, 17). Third, only strokes with more than 50% stenosis of the corresponding artery are classified as LAD (18). This means that cases with less than 50% stenosis are ignored, despite a potential to generate distal embolization. Fourth, embolic infarctions with vulnerable plaque with less than 50% stenosis are classified as SUD and aortic atherosclerosis is not described in the TOAST system even though CCS is classified as CE. Furthermore, advanced imaging techniques such as high-resolution vessel wall imaging and hemodynamic 4D-flow imaging, which can detect minimal changes in intracranial abnormalities as well as the underlying pathologic changes, can cause more confusion for classifying TOAST subtypes. Complementary classification systems are needed to resolve these problems.

**Table 1:** Characteristics of TOAST stroke subtypes.

Large artery atherosclerosis Duplex scanning or angiography must show greater than 50% stenosis of an extracranial artery relevant to lesion Cortical, cerebellar, brainstem, or subcortical hemispheric infarctions greater than 1.5 cm diameter must be present Clinical finding of cerebral cortical impairment, brainstem, or cerebellar dysfunction must be present No apparent source or suspicious cardioembolism can be present
Small vessel occlusion Patients must have a traditional lacunar syndrome of pure motor stroke, dysarthria or clumsy hand syndrome, or ataxic hemiparesis with no evidence of worsened cerebral cortical function A normal computed tomographic or magnetic resonance imaging scan or a relevant subcortical or brainstem lesion less than 1.5 cm in diameter must be present Patients must not have source of cardioembolism or stenosis of relevant extracranial artery of greater than 50%
Cardioembolism Patient must have radiologic and clinical finding similar to those seen in large artery atherosclerosis: infarctions in multiple vascular distributions are even more suggestive No relevant extracranial artery may have stenosis or greater than 50% A high- or medium-risk cardioembolic source must be identified
Other determined causes No source of cardioembolism or stenosis of a relevant extracranial artery greater than 50% can be present Other plausible sources must be identified Unknown causes Source cannot be determined, two causes are present, or evaluation is incomplete

*TOAST, Trial of ORG 10172 in Acute Stroke Treatment*.

*TOAST was the most commonly available examples of causative systems. However, with recent advances in diagnostic technology, multiple coexisting potential causes of ischemic stroke are frequently identified, which makes the selection of a causative subgroup a subjective process in clinical practice*.

In this study, we focused on the hyperintense acute reperfusion marker (HARM) in post-contrast fluid-attenuated inversion recovery (FLAIR) images in patients with acute ischemic stroke to resolve the problems of TOAST classification. The HARM is defined as a delayed enhancement of the subarachnoid or subpial space on post-contrast FLAIR images in patients with acute stroke and is believed to be associated with permeability changes in the blood–brain barrier (BBB) (19, 20). It has been reported that BBB breakdown, as indicated by HARM, was more common in patients who underwent reperfusion and was exacerbated by thrombolytic therapy (20). Recently, Ostwaldt et al. (21) demonstrated an association of HARM with early contrast material enhancement of the infarcted area in acute stroke and Lee et al. (22) reported that early HARM sign in acute stroke patients and most of HARM sign in that study was founded in the LAA and CE infarctions. They speculated that HARM may occur frequently in association with small embolic infarctions or reperfusion. We also recently observed relative high incidence of HARM in the specific cause of ischemic stroke. This study is based on *a priori* hypothesis: Embolic subtypes of acute stroke (e.g., LAA and CE) show more HARM compared with other subtypes of acute ischemic stroke based on conventional TOAST classifications. Therefore, the aims of this study were (1) to demonstrate the relationship between HARM and causative stroke etiology based on conventional TOAST classification and (2) to evaluate the relationship between HARM and stroke locations with respect to vascular territories and anatomic compartments. Based on the regions involved, HARM assessments were dichotomized into “regional pattern” and “randomized pattern.” The regional pattern described a HARM localization correlating with the same vascular territory as the acute infarction whereas the randomized pattern was assigned when a generalized contrast enhancement was detected in the ipsilateral or contralateral cerebral cortex regardless of acute infarction. Ultimately, we are going to evaluate the role of HARM for classifying stroke etiology based on TOAST classification. Understanding the clinical significance of HARM in acute stroke and incorporating these findings into the stroke subtype classification scheme may be a rational approach for acute stroke treatment and prevention.

## Materials and Methods

### Subjects and Demographic Data

#### Patient Selection

We enrolled patients admitted to our hospital between May 2015 and March 2016 who met all the following eligibility criteria: diagnosed with acute ischemic stroke; underwent conventional brain MRI including post-contrast FLAIR within 7 days of symptom onset; and showed relevant lesions upon DWI for this retrospective study. During the study period, 347 patients with acute ischemic stroke were admitted to our hospital. After excluding 79 patients due to an absence of post-contrast FLAIR and 4 patients due to a lack of information on the TOAST classification, a total of 264 patients were enrolled in this study. The study population comprised 155 male and 109 female patients with a mean age of 68.63 ± 11.14 years (range 29–92 years).

This study was approved by the institutional review board of our institute.

#### Clinical Assessment

Patient evaluation was performed by an experienced stroke neurologist. Symptom severity and neurologic recovery were assessed using the National Institute of Health Stroke Scale (NIHSS) scores at admission and discharge. We also analyzed the changes in score from admission to discharge for assessing neurologic recovery. In addition, we used modified Rankin scale (mRS) scores at discharge as a measure of clinical outcomes.

Other data such as patient demographics, medical history, and risk factors were obtained directly from the web-based stroke registry, known as the prospective stroke registry of the fifth section of the Clinical Research Center for Stroke. Clinical symptoms were classified as lacunar syndrome (including pure motor hemiparesis, pure sensory stroke, sensory-motor stroke, ataxic hemiparesis, dysarthria syndrome, and dysarthria-clumsy hand syndrome) or the non-lacunar syndrome, which was considered when cortical signs (aphasia, neglect, gaze deviation, or altered consciousness) were present. The stroke onset time was defined as the last known time at which the patient was free of symptoms. The potential stroke mechanisms were determined using the conventional TOAST classification and include presenting the atrial fibrillation as a contributing factor for cardioembolic evaluation.

### MRI Acquisition

Magnetic resonance imaging was performed on a 3 T MRI system (Achieva; Philips Medical Systems, Best, The Netherlands) equipped with an eight-channel head coil. The protocol for conventional brain MR evaluation included DWI, T2-weighted imaging, T1WI (sagittal plane), FLAIR, gradient-echo imaging, post-contrast T1WI (axial and sagittal planes), post-contrast FLAIR, and vascular imaging including three-dimensional time-of-flight (3D TOF) intracranial MRA and post-contrast MRA encompassing the extracranial internal carotid artery (ICA) and vertebral artery.

#### Post-Contrast FLAIR Imaging

Patients received gadolinium at a dose of 0.2 mmol/kg and post-contrast FLAIR images were taken 5 min later. The post-contrast FLAIR images were obtained in the axial plane using a turbo spin-echo sequence with a section thickness of 5 mm, an intersection gap of 0.5 mm, a field of view (FOV) of 182 mm × 230 mm, a TR/TE of 11,000/125 ms, and a matrix number of 352 × 176.

#### DWI Imaging

DWI was performed in the axial plane with a section thickness of 5 mm, an intersection gap of 0.5 mm, an FOV of 230 mm × 230 mm, a TR/TE of 3,000/50 ms, and a matrix number of 128 × 128. The *b* values corresponding to the diffusion-sensitizing gradient were 0 and 1,000 s/mm^2^. Apparent diffusion coefficient maps were derived automatically on a pixel-by-pixel basis from the DWI.

#### Vascular Imaging

A 3D TOF intracranial MRA was performed with a section thickness of 1.6 mm, an intersection gap of 0.8 mm, an FOV of 200 mm × 200 mm, a TR/TE of 25/3.45 ms, and a matrix number of 500 × 286. A 3D post-contrast extracranial MRA from the aortic arch was performed with a section thickness of 1 mm, an intersection gap of 0.5 mm, an FOV of 330 mm × 330 mm, a TR/TE of 5.3/2.1 ms, and a matrix number of 708 × 708.

### Image Analysis

Two experienced neuro-radiologists blinded to the patient’s clinical information performed the visual analysis of MR images.

#### Acute Infarction on DWI

The location of acute infarct was classified into subgroups according to the involved areas with respect to the vascular territory and anatomic location. The subcategories of acute infarction according to vascular territories were as follows: ICA, anterior cerebral artery, middle cerebral artery (MCA), posterior cerebral artery (PCA), basilar artery (BA), superior cerebellar artery, anterior inferior cerebellar artery, posterior inferior cerebellar artery, border zone, and multiple. The subcategories of acute infarction according to anatomic location were as follows: cortex, corona radiata, basal ganglia/internal capsule, thalamus, brainstem, and cerebellum.

#### HARM on Post-Contrast FLAIR Imaging

Hyperintense acute reperfusion marker on post-contrast FLAIR images was considered positive when cortical and sulcal contrast retention was observed. We evaluated the overall HARM incidence in patients with acute ischemic stroke in this study. We further evaluated the relative HARM incidence within each causative stroke etiology subgroup based on the conventional TOAST classification. We also accounted for the HARM pattern while evaluating the relationship between HARM and stroke etiology based on the conventional TOAST classification. The pattern of HARM was categorized by vascular territory. Based on the regions involved, HARM assessments were dichotomized into “regional pattern” and “randomized pattern.” The regional pattern described a HARM localization correlating with the same vascular territory as the acute infarction. In contrast, the randomized pattern was assigned when a generalized contrast enhancement was detected in the ipsilateral or contralateral cerebral cortex regardless of acute infarction.

### Statistical Analysis

Values are presented as mean ± SD or median (with interquartile ranges) for continuous variables and as the number of subjects (%) for categorical variables. The differences in the clinical characteristics and stroke risk factors among stroke subtypes were evaluated using the Kruskal–Wallis test for continuous variables and the Chi-square test (or Fisher’s exact test) for categorical variables. The Mann–Whitney *U*-test was used for evaluating differences in the aforementioned factors between HARM-positive and -negative groups. The differences in relative HARM incidence among stroke subtypes were evaluated using the Chi-square test (or Fisher’s exact test). In addition, for multiple comparisons, *p*-values were adjusted according to Bonferroni’s method. We also used the Chi-square test (or Fisher’s exact test) for evaluating the differences in HARM patterns among stroke subtypes. We accounted for the stroke location while evaluating the relationship between HARM and stroke etiology based on the conventional TOAST classification. The relationship between relative HARM incidence and stroke location based on vascular territory and anatomical compartments was expressed as an odds ratio (OR) with corresponding 95% confidence interval (CI) by logistic regression analysis. *p*-Values <0.05 were deemed statistically significant. All statistical analyses were performed using SPSS version 23.0 (IBM corp., Armonk, NY, USA).

## Results

### Patient Demographics and Stroke Characteristics

The sex, age, stroke risk factors, and stroke characteristics of patients are presented in Table 2.

**Table 2:** Demographics and stroke characteristics of patients according to the presence of HARM.

Variable		Total (*n* = 264)	HARM (+) (*n* = 67)		HARM (−) (*n* = 197)	*p*-Value
			Regional (*n* = 55)	Randomized (*n* = 12)		
Sex (*n*)	Male	155 (58.71%)	37 (66.07%)	6 (50%)	114 (57%)	0.367
	Female	109 (41.29%)	19 (33.93%)	6 (50%)	86 (43%)	
Age	Mean ± SD	68.63 ± 11.14	68.88 ± 10.85	71.42 ± 14.82	68.44 ± 10.97	0.301
	Median	70.50	71.00	74.00	70.00	
**Risk factors (*n*)**						
HTN (*n*)		186	40	8	140	0.971
DM (*n*)		95	21	4	71	0.872
Dyslipidemia (*n*)		176	39	7	130	0.727
Smoking (*n*)		121	29	3	90	0.793
Stroke Hx (*n*)		39	7	2	30	0.722
Large artery occlusion (*n*)		75	26	4	45	0.0006
Reperfusion therapy (*n*)		26	7	1	18	0.506
Time between symptom onset to MRI acquisition	Mean ± SD	18 h and 07 min	20 h and 33 min	21 h and 24 min	17 h and 14 min	0.118
	Median	12 h and 57 min	13 h and 5 min	15 h and 54 min	12 h and 50 min	
NIHSS at admission	Mean ± SD	4.24 ± 3.98	4.15 ± 3.14	4.83 ± 3.87	4.23 ± 4.18	0.480
	Median	4	4	4.5	4	
NIHSS at discharge	Mean ± SD	3.30 ± 3.83	3.60 ± 4.10	4.75 ± 7.29	2.13 ± 3.40	0.006
	Median	2	2	3	2	
NIHSS changes (discharge − admission)	Mean ± SD	−0.94 ± 3.80	−0.55 ± 3.83	−0.08 ± 8.76	−1.10 ± 3.23	0.053
	Median	0	0	0	0	
mRS at discharge	Mean ± SD	2.05 ± 1.31	2.13 ± 1.32	2.25 ± 1.59	2.01 ± 1.30	0.418
	Median	2	2	2	2	

*HARM, hyperintense acute reperfusion marker; HTN, hypertension; DM, diabetes; Hx, history; NIHSS, National Institute of Health Stroke Scale; MRI, magnetic resonance imaging; mRS, Modified Rankin scale*.

*Table shows the univariate associations between the demographics, stroke characteristics and the presence of HARM. There was statistically significant difference in NIHSS at discharge between HARM-positive and -negative groups. Except NIHSS at discharge, other clinical variables were similar and there were no statistically significant differences between the HARM-positive and -negative groups*.

Large artery occlusion was observed in 75 patients in total included patients and 30 patients in HARM-positive groups and 45 patients in HARM-negative group.

Twenty-four patients received intravenous (IV) tissue plasminogen activator (tPA), and two patients with cardioembolic stroke received IV tPA plus intraarterial (IA) thrombolysis. The remaining 238 patients were conservatively treated before MRI.

The mean time interval between the onset of stroke symptoms and MRI acquisition in total included patients was 18 h and 7 min (median 12 h 57 min, range 2–127 h). However, most of the patient included in current study took MRI within 48 h since symptom onset (240/264, 90%).

The median NIHSS score in total included patients at admission was 4 (range 0–28) and that at discharge was 2 (range 0–28). In addition, the median mRS score at discharge was 2 (range 0–5). However, the clinical significance of mRS scores at discharge was limited because the time interval between admission and discharge varied between 0 and 144 days (median 8 days).

In this study population, SVO was the most common cause of acute ischemic stroke (33.3%), followed by LAA (28.4%), SUD (25.0%), CE (11.7%), and SOD (0.02%), based on the conventional TOAST classification. Among the SUD cases (*n* = 66), negative causes were the most common etiology (*n* = 40), followed by two or more causes (*n* = 15), and incomplete work-ups (*n* = 11).

With respect to stroke location according to the involved vascular territory, MCA was the most frequently involved vascular territory of the stroke location (*n* = 164), followed by PCA (*n* = 45) and BA (*n* = 37). With respect to stroke location based on anatomic compartments, DWI demonstrated acute ischemic infarction in the cortex in 122 patients, in the corona radiata in 86 patients, in the basal ganglia/internal capsule in 89 patients, in the thalamus in 30 patients, in the brainstem in 32 patients, and in the cerebellum in 19 patients.

### Relationship between HARM and Causative Stroke Etiology Based on Conventional TOAST Classification

**Figure 1:**
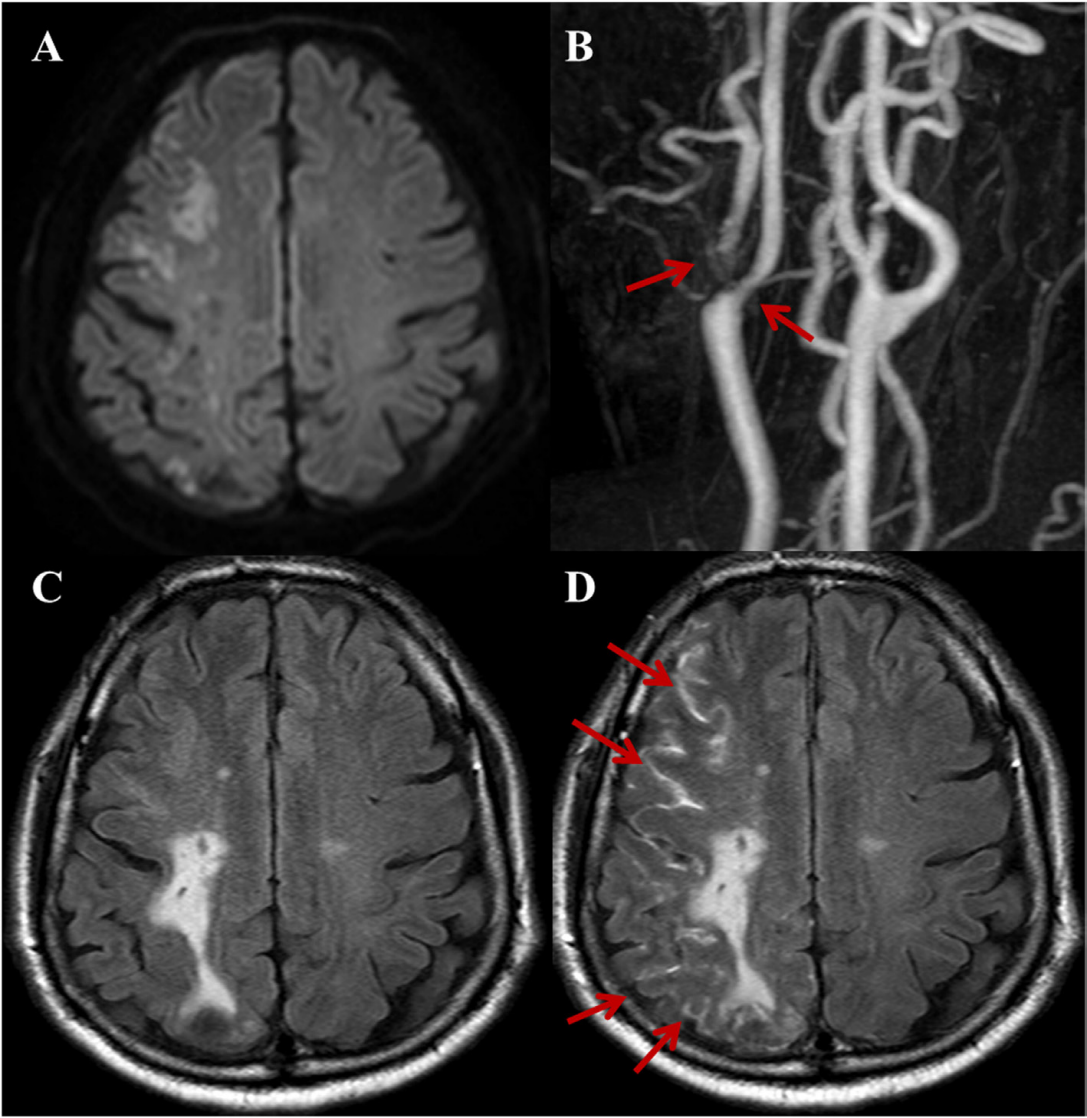
A 72-year-old male with a complaint of acute onset left arm weakness. Stroke due to large artery to artery atherosclerosis. **(A)** DWI shows acute border zone infarctions on the right cerebral hemisphere. **(B)** Three-dimensional time-of-flight MRA of extracranial arteries shows moderate-to-severe stenosis with plaque at right proximal internal carotid artery and ECA (arrows). Compared with non-contrast fluid-attenuated inversion recovery (FLAIR) **(C)**, the post-contrast FLAIR **(D)** image shows diffuse sulcal enhancement (hyperintense acute reperfusion marker sign) along the right cerebral sulci (arrows).

**Figure 2:**
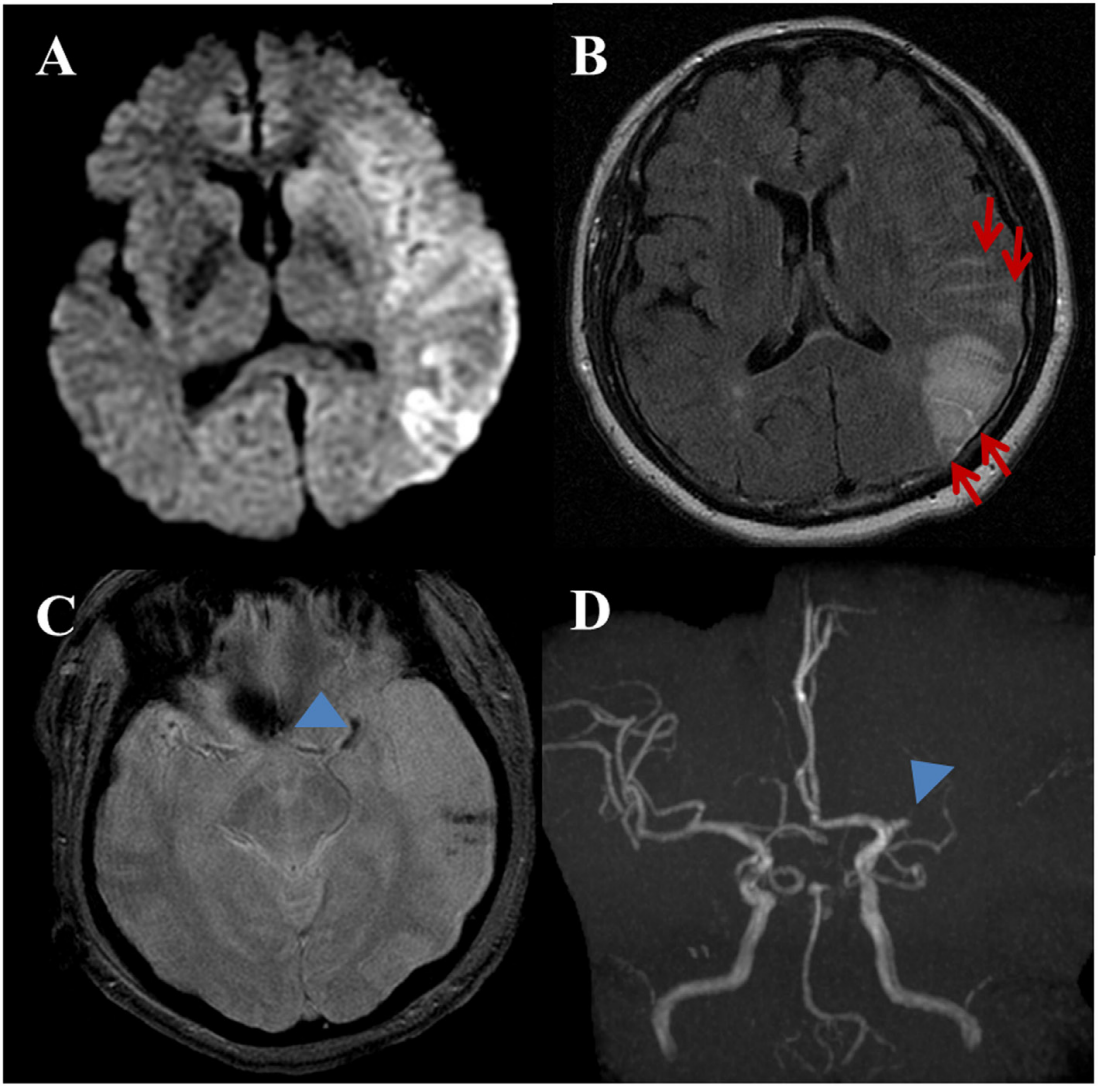
A 53-year-old female with a complaint of acute onset aphasia. Stroke due to cardioembolic. **(A)** DWI shows large diffusion restricted lesion on the left frontoparietotemporal lobes. **(B)** Post-contrast fluid-attenuated inversion recovery image shows sulcal enhancement along the left frontoparietal sulci (arrows) compatible with hyperintense acute reperfusion marker. **(C)** Gradient-echo imaging image shows blooming artifact at the left proximal MCA (arrowhead) indicating intravascular thrombus. Note, hemorrhagic foci of stroke area at the left temporal lobe. **(D)** Three-dimensional time-of-flight MRA shows occlusion of proximal M1 segment of left MCA (arrowhead). There is no stenoocclusive lesion in the cervical arteries (not shown).

**Figure 3:**
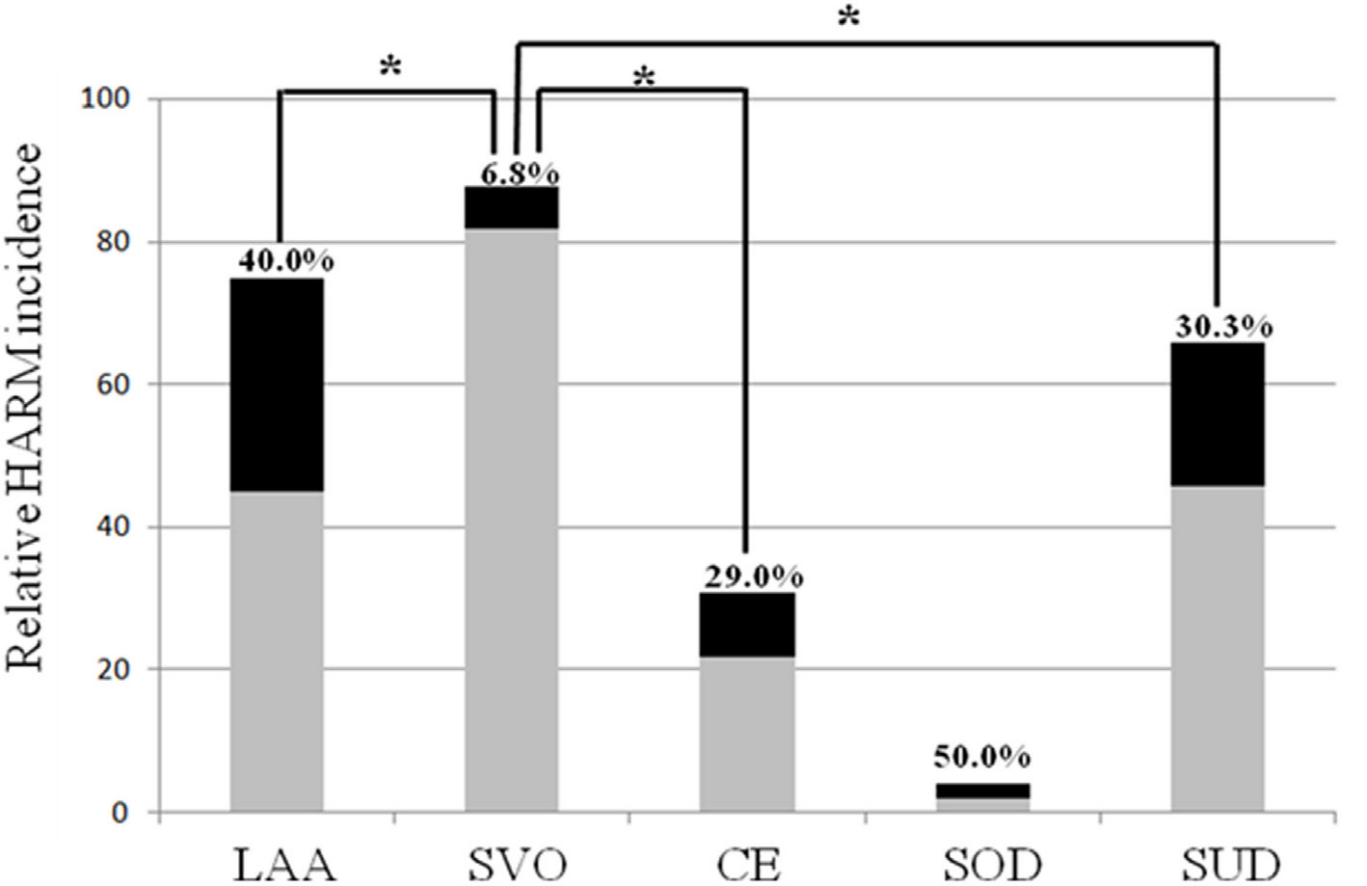
Multiple comparisons of the relative hyperintense acute reperfusion marker (HARM) incidence according to stroke subtypes.

Among the 264 patients with acute ischemic stroke, 67 (25.38%) patients were HARM-positive while and 197 (74.62%) patients were HARM-negative (Table 3A). We evaluated the relative HARM incidence between subgroups of stroke etiology based on the conventional TOAST classification: there was a significant difference in relative HARM incidence among the stroke subgroups (*p* < 0.001) (Table 3A). Table 3B shows the relatively higher incidence of HARM in the LAA group (40%) than in the other subgroups (19.57%) (*p* = 0.001). In addition, the relative HARM incidence in LAA plus CE (36.79%) subgroup was significantly higher compared to that in the other subgroups (17.72%) (*p* < 0.001) (Table 3C; Figures 1 and 2). Table 3D shows a significantly lower incidence of HARM in the SVO subgroup (6.81%) than in the other subgroups (34.65%) (*p* < 0.001). The relative HARM incidence was significantly different between the LAA and SVO (*p* = 0.001), SVO and CE (*p* = 0.016), and SVO and SUD subgroups (*p* = 0.001) (Table 4; Figure 3).

**Table 3:** Relative HARM incidence of patients according to stroke subtypes.

	TOAST								*p*-Value		
	Total (*n* = 264)	1. LAA (*n* = 75)	2. SVO (*n* = 88)	3. CE (*n* = 31)	4. SOD (*n* = 4)	5. SUD (*n* = 66)			Among subtypes	LAA vs. other subtypes	SVO vs. other subtypes
		
						SUDm (*n* = 15)	SUDn (*n* = 40)	SUDi (*n* = 11)			
**(A) OVERALL TOAST ETIOLOGIC SUBGROUPS**											
HARM (+)	67	30	6	9	2	3	13	4	<0.0001	0.001	<0.0001
HARM (−)	197	45	82	22	2	12	27	7			
**(B) LAA VS. OTHER SUBGROUP**											
	**TOAST**										
	**LAA (*n* = 75)**	**Others (*n* = 189)**	***p*-Value**								
								
HARM (+) (*n* = 67)	30 (40%)	37 (19.57%)	0.001								
HARM (−) (*n* = 197)	45 (60%)	152 (82.42%)									
**(C) LAA PLUS CE VS. OTHER GROUP**											
	**TOAST**										
	**CE + LAA (*n* = 106)**	**Others (*n* = 158)**	***p*-Value**								
								
HARM (+) (*n* = 67)	39 (36.79%)	28 (17.72%)	<0.001								
HARM (−) (*n* = 197)	67 (63.21%)	130 (82.28%)									
**(D) SVO VS. OTHER SUBGROUPS**											
	**TOAST**										
	**SVO (*n* = 88)**	**Others (*n* = 176)**	***p*-Value**								
								
HARM (+) (*n* = 67)	6 (6.81%)	61 (34.65%)	<0.001								
HARM (−) (*n* = 197)	82 (93.18%)	115 (65.34%)									

*HARM, hyperintense acute reperfusion marker; TOAST, Trial of ORG 10172 in Acute Stroke Treatment; LAA, large artery atherosclerosis; SVO, small vessel occlusion; CE, cardioembolism; SOD, strokes of other determined etiology; SUD, strokes of undetermined etiology; SUDm, two or more causes; SUDn, negative evaluation; SUDi, incomplete evaluation*.

*Among the 264 patients with acute ischemic stroke, 67 (25.38%) patients were HARM positive while, 197 (74.62%) patients were HARM negative (A). (B) The relatively higher incidence of HARM in the LAA group (40%) than in the other subgroups (19.57%) (*p* = 0.001). (C) The relative HARM incidence in LAA plus CE (36.79%) subgroup was significantly higher compared with that in the other subgroups (17.72%) (*p* < 0.001). (D) A significantly lower incidence of HARM in the SVO subgroup (6.81%) than in the other subgroups (34.65%) (*p* < 0.001)*.

**Table 4:** Multiple comparisons of the relative HARM incidence according to stroke subtypes.

	LAA vs. SVO	LAA vs. CE	LAA vs. SOD	LAA vs. SUD	SVO vs. CE	SVO vs. SOD	SVO vs. SUD	CE vs. SOD	CE vs. SUD	SOD vs. SUD
*p*-Value	0.011*	1.000	1.000	1.000	0.016**	0.366	0.001***	1.000	1.000	1.000

*HARM, hyperintense acute reperfusion marker; LAA, large artery atherosclerosis; SVO, small vessel occlusion; CE, cardioembolism; SOD, strokes of other determined etiology; SUD, strokes of undetermined etiology*.

*Table shows the relative HARM incidence was significantly different between the LAA and SVO (*p* = 0.001), SVO and CE (*p* = 0.016), and SVO and SUD (*p* = 0.001) subgroups*.

**Figure 4:**
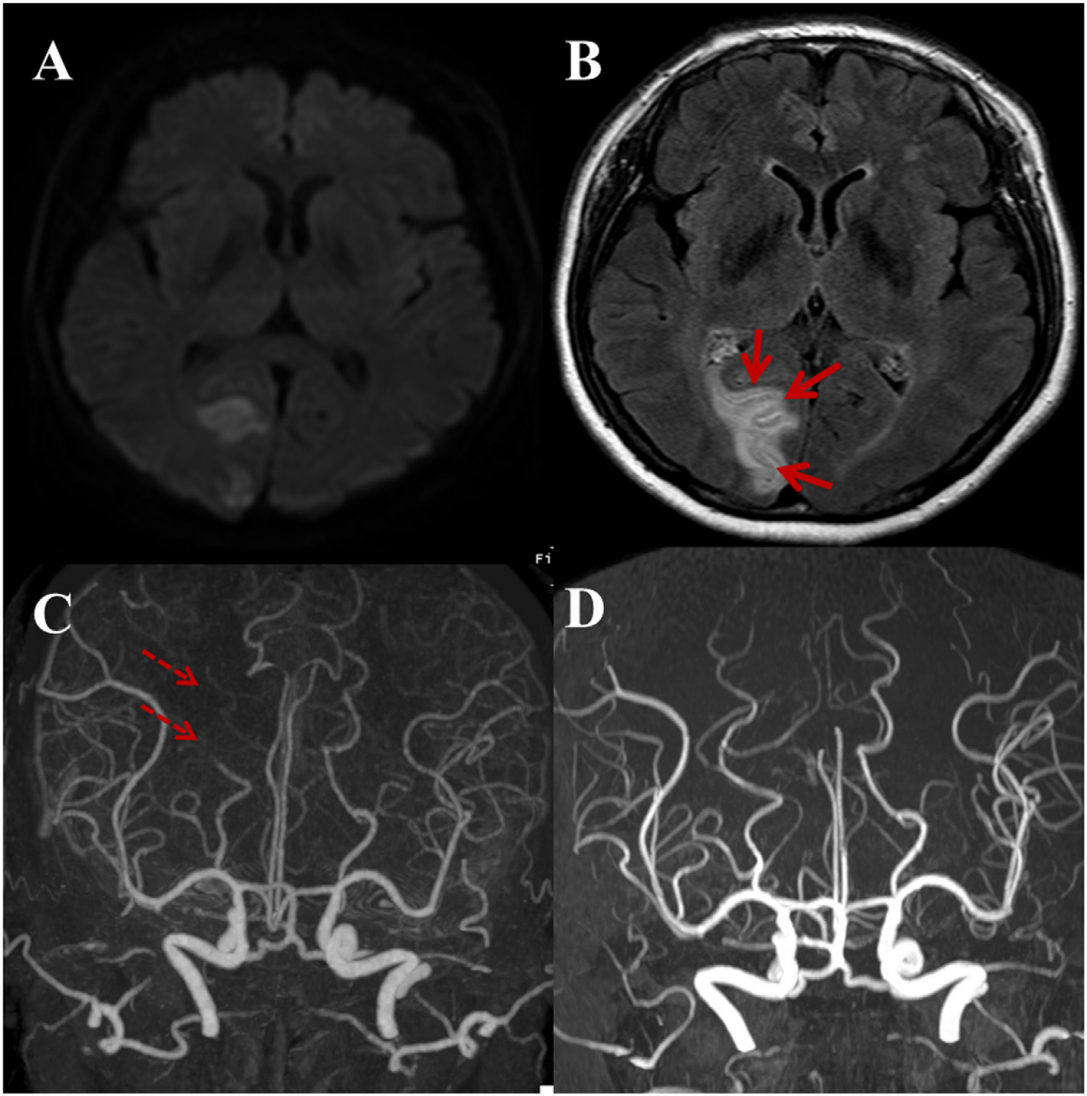
A 59-year-old male with a complaint of acute onset left side visual field defect in both eyes. Regional pattern of hyperintense acute reperfusion marker. **(A)** DWI shows acute infarction at the right occipital lobe involving the visual cortex. **(B)** Post-contrast fluid-attenuated inversion recovery image shows sulcal enhancement along the right occipital lobe (arrows). Compared with outside CTA **(C)**, three-dimensional time-of-flight MRA **(D)** shows the recanalization state of right distal posterior cerebral artery (dashed arrows).

**Figure 5:**
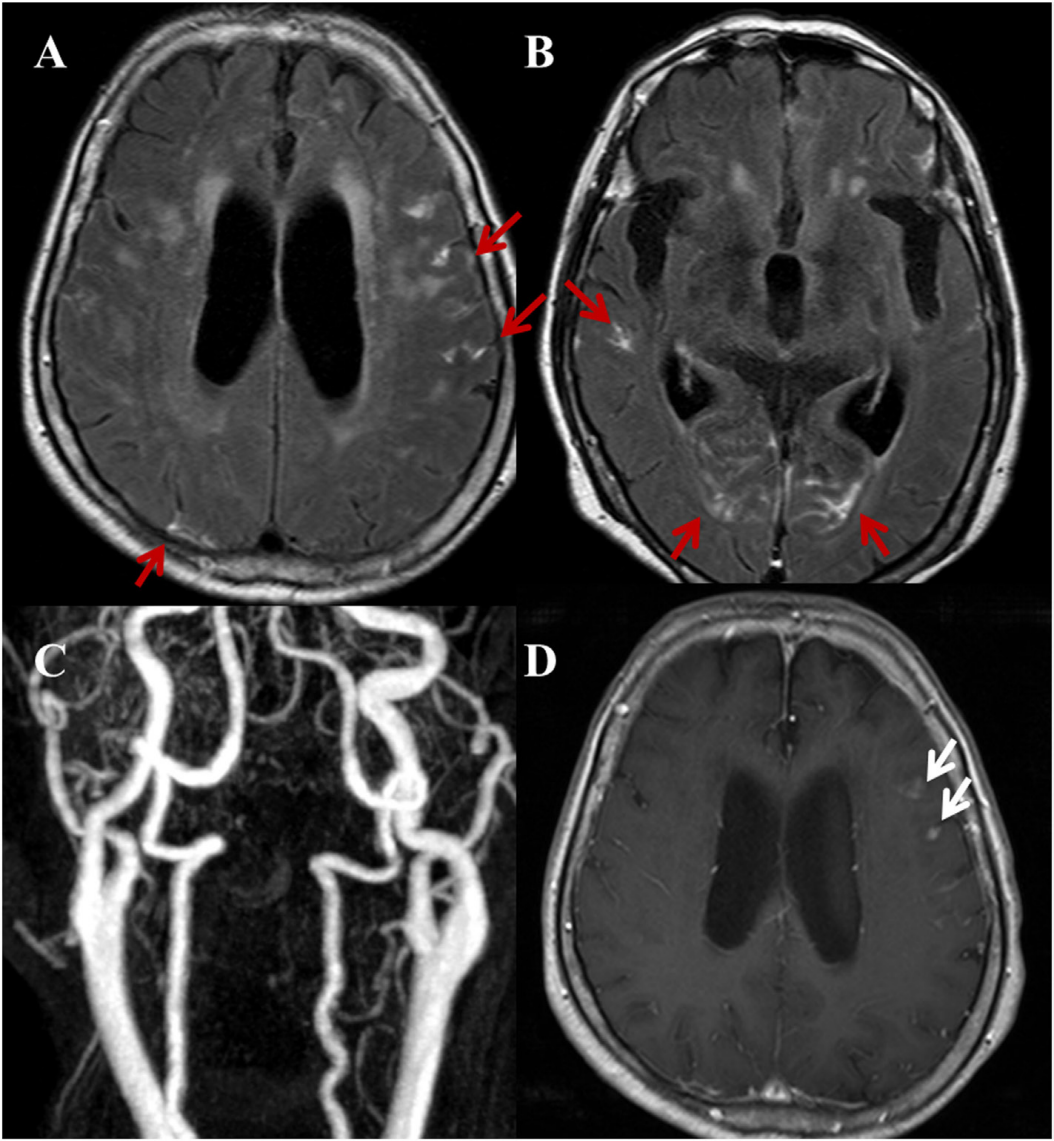
A 82-year-old female with a complaint of acute onset dizziness. Randomized pattern of hyperintense acute reperfusion marker. **(A,B)** Post-contrast fluid-attenuated inversion recovery images show a diffuse, multifocal sulcal enhancement along both cerebral hemispheres (red arrows). **(C)** Three-dimensional time-of-flight MRA of cervical arteries does not show a stenoocclusive lesion. Furthermore, there was no stenoocclusive lesion in the intracranial arteries (not shown). **(D)** Contrast-enhanced T1WI shows multiple nodular or dot-like enhancing lesions (white arrows) on both cerebral hemispheres (not fully shown in this image) indicating acute to subacute stage infarctions.

Of the 67 patients in the HARM-positive group, 55 patients showed a regional pattern of HARM with linear enhancement on the same vascular territory as the acute infarction, whereas 12 patients showed a randomized pattern of HARM with linear enhancement on multiple vascular territories in the ipsilateral or contralateral cerebral cortex regardless of acute infarction (Figures 4 and 5). Table 5 shows the relative HARM incidences and HARM patterns between subgroups of stroke etiology. There was a statistically significant difference in HARM patterns among different subtypes (*p* < 0.001). LAA showed a significant association with the regional HARM pattern, compared with the other subgroups (*p* = 0.002).

**Table 5:** Relative HARM incidence and HARM patterns of patients according to stroke subtypes.

	TOAST								*p*-Value		
	Total (*n* = 264)	1. LAA (*n* = 75)	2. SVO (*n* = 88)	3. CE (*n* = 31)	4. SOD (*n* = 4)	5. SUD (*n* = 66)			Among subtypes	LAA vs. other subtypes	SVO vs. other subtypes
						SUDm (*n* = 15)	SUDn (*n* = 40)	SUDi (*n* = 11)			
HARM (+)	67	30	6	9	2	3	13	4			
Regional	55	26	3	8	1	2	12	3	<0.0001	0.002	<0.0001
Randomized	12	4	3	1	1	1	1	1			
HARM (−)	197	45	82	22	2	12	27	7			

*HARM, hyperintense acute reperfusion marker; LAA, large artery atherosclerosis; SVO, small vessel occlusion; CE, cardioembolism; SOD, strokes of other determined etiology; SUD, strokes of undetermined etiology; SUDm, two or more causes; SUDn, negative evaluation; SUDi, incomplete evaluation; TOAST, Trial of ORG 10172 in Acute Stroke Treatment*.

*Table shows the relative HARM incidences and HARM patterns between subgroups of stroke etiology. There was a statistically significant difference in HARM patterns among different subtypes (*p* < 0.001). LAA showed a significant association with the regional HARM pattern, compared with the other subgroups (*p* = 0.002)*.

Table 6 shows the univariate associations between the clinical variables and the presence of HARM. All clinical variables were similar, and there were no statistically significant differences between the HARM-positive and -negative groups.

**Table 6:** Relative HARM incidence of patients according to stroke location.

	Total	HARM (+)		HARM (−)	Logistic regression			
		Regional	Randomized		*p*-Value	Odds ratio	CI	
**(A) Stroke location by vascular territories**								
ICA	7 (2.61%)	2 (3.57%)	0 (0%)	5 (2.5%)	0.844	1.182	0.224	6.237
MCA	165 (61.57%)	41 (73.21%)	8 (66.67%)	116 (58%)	0.041	1.868	1.025	3.401
ACA	13 (4.85%)	3 (5.36%)	0 (0%)	10 (5%)	0.846	0.877	0.234	3.285
PCA	45 (16.79%)	12 (21.43%)	2 (16.67%)	31 (15.5%)	0.334	1.413	0.701	2.851
BA	37 (13.81%)	1 (1.79%)	0 (0%)	36 (18%)	0.009	0.068	0.009	0.506
VA	4 (1.49%)	0 (0%)	0 (0%)	4 (2%)	0.985	<0.001	<0.001	>999.999
SCA	5 (1.87%)	1 (1.79%)	0 (0%)	4 (2%)	0.781	0.731	0.080	6.659
AICA	3 (1.12%)	0 (0%)	0 (0%)	3 (1.5%)	0.987	<0.001	<0.001	>999.999
PICA	17 (6.34%)	3 (5.36%)	2 (16.67%)	12 (6%)	0.693	1.243	0.422	3.667
Border zone	8 (2.99%)	4 (7.14%)	0 (0%)	4 (2%)	0.121	3.063	0.744	12.598
Multiple	28 (10.45%)	9 (16.07%)	0 (0%)	19 (9.5%)	0.386	1.453	0.624	3.386

	**Total**	**HARM (+)**		**HARM (−)**	**Logistic regression**			
		**Regional**	**Randomized**		***p*-Value**	**Odds ratio**	**CI**	

**(B) Stroke location by anatomic compartments**								
Cortex	122 (45.52%)	50 (89.29%)	6 (50%)	66 (33%)	<0.0001	9.475	4.754	18.883
CoronaR	86 (32.09%)	16 (28.57%)	5 (41.67%)	65 (32.5%)	0.805	0.928	0.513	1.680
BG + IC	89 (33.21%)	15 (26.79%)	4 (33.33%)	70 (35%)	0.287	0.720	0.394	1.318
Thalamus	30 (11.19%)	7 (12.5%)	1 (8.33%)	22 (11%)	0.863	1.079	0.456	2.551
Brainstem	32 (11.94%)	4 (7.14%)	0 (0%)	28 (14%)	0.084	0.384	0.130	1.138
Cerebellum	19 (7.09%)	3 (5.36%)	2 (16.67%)	14 (7%)	0.921	1.055	0.365	3.046

*HARM, hyperintense acute reperfusion marker; ICA, internal carotid artery; MCA, middle cerebral artery; ACA, anterior cerebral artery; PCA, posterior cerebral artery; BA, basilar artery; VA, vertebral artery; SCA, superior cerebellar artery; AICA, anterior inferior cerebellar artery; PICA, posterior inferior cerebellar artery; CI, confidence interval; coronaR, corona radiate; BG, basal ganglia; IC, internal capsule*.

*Table shows the relative HARM incidence between the HARM-positive and -negative groups according to stroke location by the involved vascular territory and anatomic compartment*.

### Relationship between HARM and Stroke Location with Respect to Vascular Territory and Anatomic Compartment

Table 6 shows the relative HARM incidence between the HARM-positive and -negative groups according to stroke location by the involved vascular territory and anatomic compartment. In terms of vascular territory, the relative HARM incidence in MCA and BA showed a statistically significant difference with *p*-values of 0.041 and 0.009, respectively. The OR for HARM from MCA infarction was 1.868 (95% CI: 1.025–3.401) (Table 6A), indicating that MCA infarction is associated with a higher incidence of HARM than infarction at other vascular territories. In contrast, the relative HARM incidence in BA showed a negative correlation with an OR of 0.068 (95% CI: 0.009–0.506).

However, for different anatomic compartments, the OR for HARM from cortical infarction was 9.475 (95% CI: 4.754–18.883) (Table 6B), indicating that cortical infarction is associated with a significantly higher incidence of HARM than stroke in other anatomic compartments.

## Discussion

In the current study, relative HARM incidence was significantly higher in the LAA subgroup than in the other subgroups (*p* = 0.001), while the SVO subgroup showed a relatively lower HARM incidence compared with the other subgroups (*p* < 0.001). Although CE itself showed no statistically significant difference in HARM incidence compared with the other etiologic subgroups, the relative HARM incidence in the overall embolic infarctions, including the LAA and CE subgroups, was higher compared with that in the other subgroups (*p* < 0.001). In terms of stroke location, the relative HARM incidence in the MCA territorial infarction and cortical infarction subgroups was higher than that in other vascular and anatomic territories. These findings suggest that HARM on post-contrast FLAIR images is associated with specific stroke causes, especially embolic infarction.

HARM is well known to represent the BBB permeability in ischemic stroke patients (19, 20). Ischemic stroke evokes a marked biphasic opening of the BBB. Early opening of the BBB takes place within the first 30 min of reperfusion and is followed by partial closing. The initial acute opening is described as a “hemodynamic” BBB opening resulting in cytotoxic edema (23, 24). Subsequently, a second delayed opening occurs 24–48 h later after reperfusion; this second opening results in vasogenic edema and is induced by “inflammation” (25). In the early opening phase, ischemic stroke restricts the delivery of oxygen and glucose to the brain tissue, which leads to a cascade of ischemic events and subsequent decrease in blood flow and ATP levels. Consequently, metabolic failure, ionic imbalance, and finally cellular death progresses within minutes (26). Following this, activated astrocytes and pericytes participate in the inflammatory cascade and release a number of substances that may be harmful to brain tissue (27). This wide variety of responses results in a number of effects including the loss of tight junctions between endothelial cells and finally results in the increase in BBB permeability (28).

We believe that HARM is more visible in the embolic infarctions, as compared with other TOAST subtypes, because of a lack of tolerance to the sudden hemodynamic changes in embolic infarction cases. High ischemic tolerance has been noted in atherosclerotic thromboembolic infarction rather than in embolic infarction and in old age patients rather than in young age stroke patients. Furthermore, the high recanalization rate in cardioembolic infarction is attributable to HARM incidence. In 4.7–12% of cases, cardioembolic infarction showed a sudden onset of neurologic deficit followed by dramatic improvement of the initially severe neurologic symptoms. This may be due to distal migration of the embolus followed by recanalization of the occluded vessel (29). These sudden hemodynamic changes might contribute to the relatively higher incidence of the HARM in embolic infarction.

There are other findings to back up these theories in the current study. Within the LAA etiology group (*n* = 75), 30 patients were HARM positive and the remaining 45 patients were HARM negative. We speculate that these results may be due to different pathophysiological mechanisms. The pathophysiological mechanisms of LAA have been proposed as (1) hypoperfusion of the involved vascular territory, (2) unstable plaque with artery-to-artery embolism, and (3) hypoperfusion-mediated microembolus formation. It is difficult to infer which mechanism may be responsible for an individual patient’s condition in clinical practice (30). We hypothesized that the presence of HARM in acute stroke patients diagnosed with LAA etiology may be attributable to the artery-to-artery embolism, whereas the absence of HARM may be attributable to hypoperfusion.

Other studies have also found a higher incidence of HARM in MCA territorial infarction and cortical infarction compared to infarction at other vascular territories and anatomic compartments. Using high-resolution phase-contrast MRI, most of the IA blood flow was found to be distributed to the MCA (31). We hypothesized that the higher incidence of HARM in MCA territorial infarction may be attributable to the embolic stroke etiology in terms of IA flow dynamics. This result is in agreement with the findings of Garcia et al. (32), which show that most emboli lodge in the MCA distribution because 80% of the blood carried by large neck arteries flows through the MCA. Regarding stroke location according to the involved anatomic compartment, our study demonstrated a higher incidence of HARM with cortical infarction. This result is also in line with a previous study in which acute stroke lesions in the cortex and subcortical area were associated with CE and LAA infarction (33).

However, there were no significant differences in the relative HARM incidence between the CE subgroup itself and other etiologic subgroups, contrary to our priori hypothesis. This result could be explained by the relatively high proportion of “undetermined etiology” cases in the current study. Under the conventional TOAST classification, the presence of atrial fibrillation combined with other possible stroke etiologies belongs to the category of “undetermined etiology.” However, we speculated that the different histological compositions of the cardiac origin emboli and artery-to-artery emboli from large artery disease could have produced the differences in relative HARM incidence between artery-to-artery emboli and cardiogenic emboli. Emboli of cardiac origin are derived from blood stasis in the posterior left atrium and appendage and are mainly composed of fibrin and red blood cells (RBCs). In contrast, large artery-to-artery emboli are derived from atheromatous plaques in the aorta and carotid artery at the site of ulcerated plaque and are mainly composed of platelets and fibrin (34, 35). We hypothesized that large artery-to-artery emboli are more likely to induce BBB disruption compared to cardiac emboli, as RBC-rich emboli are promptly degraded by an intrinsic thrombolytic cascade. Notably, the composition of emboli may play a role in the success rate of thrombolysis with tPA since RBC-rich thrombi are known to be more vulnerable to thrombolysis compared to platelet-rich thrombi (36, 37).

In the current study, the majority of the SVO etiologic groups did not show HARM (*p* < 0.001). In a previous study, BBB dysfunction, indicated by IV injected contrast leakage, was demonstrated in white matter disease and dementia (38, 39), as well as in lacunar infarction (40). However, in the current study, BBB disruption, as indicated by HARM, was relatively low in the SVO etiologic group. In terms of our hypothesis that embolic sources of acute infarction cause more HARM than other stroke etiologies, our results are in line with that of a previous study, which demonstrated that cardiac or artery-to-artery embolism is unlikely to be a frequent cause of most lacunar strokes (41).

In contrast, among the SVO etiologic group (*n* = 88), six patients showed HARM signs. This finding may be attributable to other causes or TIA rather than acute lacunar infarction itself. Recent studies have shown that TIA and imaging-negative strokes might be HARM positive and associated with focal BBB disruption (40, 42). Lee et al. (43) suggested that a finding of HARM without diffusion abnormalities represents a specific type of TIA with favorable short-term neurologic outcomes, compared with imaging-negative stroke. The prevalence of SVO may be artifactually elevated to a misclassification of stroke subtypes (18). SVO usually presents as single subcortical infarction. Furthermore, recent studies have shown that single subcortical infarctions frequently are caused by branch occlusion associated with parental artery atherosclerotic plaque, arteriosclerotic proximal small vessel disease, or other embolic sources, rather than lipohyalinotic small artery disease (44).

We analyzed the topographic pattern of HARM with regard to stroke locations. Of the 67 patients in the HARM-positive group, 55 patients showed a linear cortical or sulcal enhancement along the same vascular territory near the acute infarction (regional pattern), whereas 12 patients showed ipsilateral or contralateral enhancement regardless of the acute infarction vascular territory (randomized pattern). Of note, LAA showed a significantly higher association with the regional HARM pattern relative to the other subgroups (*p* = 0.002). Anatomically, the perivascular space is filled with interstitial fluid or CSF, encompassing the surface and penetrating the blood vessels of the brain. It has been demonstrated that systolic arterial pulsation is derived from CSF motion in the perivascular space (45). We speculate that this pulsatile CSF motion, termed “extracerebral CSF dynamic,” may influence the extent of contrast spillage indicated by the HARM pattern.

Blood–brain barrier opening indicated by a HARM sign is distinct from enhancement of the leptomeninges (46, 47) and from the parenchyma enhancement that is observed during the subacute stage of cerebral ischemia (48). A recent study reported that acute ischemic stroke patients with HARM showed a higher relative signal intensity compared with HARM-negative patients on FLAIR images in the adjacent brain parenchyma corresponding to the DWI-positive tissue, which is likely due to parenchymal enhancement (21). This result indicates that HARM does not only represent a contrast leakage from the pial system into the CSF space. However, it is not yet clear whether HARM is also associated with extravasation of contrast agent into the parenchyma.

In previous HARM studies, the commonly used gadolinium-containing contrast agents were Magnevist (20, 49) or Gadovist (50). In the present study, we administered Gadovist at a dose of 0.2 mmol/kg. Ostwaldt et al. (51) revealed that increasing the dosage of Gadobutrol is also associated with increased HARM incidence in acute stroke patients. Post-contrast FLAIR images obtained at 5 min after contrast administration showed what was described as “early” HARM in a previous study (22). It should be emphasized that these “early HARM” signs are different from those of other previous studies in which HARM was depicted on follow-up unenhanced FLAIR images at 2–48 h after contrast administration in the initial MRI. In the current study, HARM was detected in 25.38% of acute ischemic strokes compared to the 5.5% reported by Lee et al. (22). This discrepancy might be attributable to the treatments applied before MRI image acquisition in the present study. In addition, the interval between symptom onset and MRI acquisition varied from 0 h to 7 days in the current study compared to 0–24 h in the study by Lee et al. (22). However, the incidence of HARM evaluated at 2–48 h after contrast administration was 33–43% (20, 49, 50).

Although the HARM and risk of hemorrhagic transformation associated with sudden hemodynamic changes after carotid artery stenting or temporary balloon occlusion of the carotid artery have been widely studied in previous studies, there is little information on the relationship between the HARM and stroke etiology. The current study demonstrated that the HARM is associated with embolic infarction. This hypothesis is supported by our findings of a relatively higher incidence of the HARM in MCA and cortical infarction compared with infarction at other vascular territories and anatomic compartments. The results from this study may be translated into clinical practice and management, suggesting that evaluation of embolic sources needs to be done thoroughly, especially in HARM-positive strokes. It has been demonstrated that the HARM is not a specific radiologic finding in acute stroke and can be understood as a common finding in other BBB-disrupting conditions, such as postcarotid stent, postcardiac surgery, and traumatic brain injury (22). However, understanding the clinical significance of HARM signs in acute stroke and incorporating these findings into a stroke subtype classification scheme may be a rational approach for acute stroke treatment and prevention. Moreover, strict application of the conventional TOAST criteria could lead to categorization of the majority of strokes in the “undetermined etiology” category (52). Elucidating the most likely mechanism among coexisting potential stroke causes would therefore be useful. We expect that with the recent advances in stroke imaging, including the HARM on post-contrast FLAIR, it is possible to establish the correct classification of stroke.

With recent advancements in imaging, high-resolution MRIs can detect small plaques causing infarction, even in patients with normal vascular findings, which would not be classified as LAA etiology under previous classification systems (18). Despite the practical issues in using MRI for acute stroke evaluation, MRI can be useful for the accurate etiologic diagnosis of not only stroke but also stroke prognosis.

This study has a number of limitations. First, the sample population may be biased, as it is a retrospective single-center study from a single region. This cohort has relatively mild stroke with median NIHSS around 4–5. Moreover, the distribution of stroke subtypes in our study showed a higher incidence of SVO than the population average in Korea (18). Second, symptom onset varied between the early hyper-acute (0–6 h), late hyper-acute (6–24 h), and acute (24 h to 1 week). The third drawback of this study is the limited evaluation of the most probable cause of stroke etiology in the undetermined group based on conventional TOAST classification. Numerous patients in the current study had a stroke of undetermined etiology, comprising up to 25%. Fourth, IV tPA and IA thrombolysis were performed in very few patients at varied timings; therefore, we could not identify the relationship between the HARM and applied treatments. Fifth, the infarct volume is not supplemented due to we included both single and multiple infarctions in this study. Further studies employing larger datasets with prospective study design and pathologic confirmation are needed to confirm the results in current study.

In conclusion, the presence of the HARM was significantly associated with embolic infarctions including LAA and CE. Conversely, SVO was exclusively associated with the absence of the HARM. Second, MCA and cortical infarction showed a more pronounced HARM compared to infarctions at other vascular territories and anatomic compartments. According to the results in the current study, we speculate that the presence of HARM on post-contrast FLAIR images was associated with specific stroke causes especially in embolic causes. However, future studies are required to confirm these preliminary findings and to determine if HARM can provide independent information on stroke causes.

## Ethics Statement

This retrospective study was approved by our institutional review board. Informed consent was waived because only imaging findings and demographic data were retrieved from the picture archiving and communication system (PACS) server and medical records.

## Author Contributions

HC and H-GK collected the data and wrote the article. KL conceived and designed the study and wrote the article. EK, WC, BK, SH, and D-IC critically revised the article for important intellectual content.

## Conflict of Interest Statement

The authors declare that the research was conducted in the absence of any commercial or financial relationships that could be construed as a potential conflict of interest.
